# Update on Hepatitis C Vaccine: Results and Challenges

**DOI:** 10.3390/v16081337

**Published:** 2024-08-21

**Authors:** Anna Rosa Garbuglia, Silvia Pauciullo, Verdiana Zulian, Paola Del Porto

**Affiliations:** 1 Laboratory of Virology, National Institute for Infectious Diseases “Lazzaro Spallanzani” (IRCCS), 00149 Rome, Italy; silvia.pauciullo@inmi.it (S.P.); verdiana.zulian@inmi.it (V.Z.); 2 Department of Biology and Biotechnology “Charles Darwin”, Sapienza University of Rome, 00100 Rome, Italy; paola.delporto@uniroma1.it

**Keywords:** hepatitis C, vaccine, immune response, immunogenicity, neutralizing antibody

## Abstract

Therapy against the Hepatitis C virus (HCV) has significantly improved with the introduction of direct-acting antiviral drugs (DAAs), achieving over 95% sustained virological response (SVR). Despite this, the development of an effective anti-HCV vaccine remains a critical challenge due to the low number of patients treated with DAAs and the occurrence of HCV reinfections in high-risk groups. Current vaccine strategies aim to stimulate either B-cell or T-cell responses. Vaccines based on E1 and E2 proteins can elicit broad cross-neutralizing antibodies against all major HCV genotypes, though with varying efficiencies and without full protection against infection. In humans, the neutralizing antibodies induced by such vaccines mainly target the AR3 region, but their levels are generally insufficient for broad neutralization. Various HCV proteins expressed through different viral vectors have been utilized to elicit T cell immune responses, showing sustained expansion of HCV-specific effector memory T cells and improved proliferation and polyfunctionality of memory T cells over time. However, despite these advancements, the frequency and effectiveness of T-cell responses remain limited.

## 1. Introduction

The Hepatitis C virus (HCV) infects more than 185 million people worldwide [1]. Health complications such as cirrhosis, liver failure, and hepatocellular carcinoma (HCC) linked to chronic HCV infection cause around 580,000 deaths per year [2,3]. HCC is caused by the direct activity of viral proteins and the indirect effects of fibrinogenesis, dysfunctional immunity, and chronic liver inflammation [4]. HCV is considered the main risk factor for HCC in developed countries [5]. Overall, HCC accounts for approximately 75% of total liver cancers, with 31% of them being HCV-positive [6]. Prevention of the onset of HCC is hampered by the low diagnosis rate of HCV infection, wherein over 79% of infections remain undiagnosed [7]. Rapid diagnostic tests (RDTs) represent a valid instrument for screening HCV subjects; however, their cost and the lack of infrastructure for follow-up and treatment of HCV-positive subjects represent an obstacle to the care and management of HCV patients, especially in low-income countries.

HCV typically progresses slowly, with persistent hepatic inflammation often resulting in cirrhosis in about 10–20% of patients within 20–30 years. Nonetheless, there is substantial variability in the rate of progression to cirrhosis, ranging from 2–3% to 51% over 22 years [8,9]. Recent research has highlighted that the progression of hepatic fibrosis in hepatitis C is influenced by multiple factors. Various elements have been identified that increase the risk of developing significant fibrosis or cirrhosis, including the age at which the infection occurs, male gender, alcohol use, obesity, insulin resistance, type 2 diabetes, co-infection with hepatitis B or HIV, use of immunosuppressive therapy, and genetic predispositions. Additionally, factors affecting fibrosis progression can evolve over time, indicating that the advancement of hepatitis C is not necessarily a linear process [10,11,12]. Genetic polymorphisms have been associated with progression towards chronic HCV infection. Polymorphisms in the TNFA gene, such as TNFA-308G- > A and IL10-1082A- > G, appear to be predictive biomarkers of chronic HCV infection [13]. Additionally, the IL28B gene polymorphism rs12979860, but not rs8099917, contributes to the occurrence of chronic HCV infection in Uruguayan patients [14].

Therapy against HCV has been considerably improved by the introduction of direct-acting antiviral drugs (DAAs) with pangenotypic efficacy, through which the success of therapy reaches over 95% of sustained virological response (SVR) [15,16]. These drugs are inhibitors of three different viral proteins: NS3/4A protease, NS5A, and NS5B polymerase [17]. Because these DAAs target three different viral proteins, any form of resistance to one antiviral drug can be neutralized by sensitivity to another.

However, the development of an anti-HCV vaccine remains a crucial issue for several reasons: (1) the low number of patients treated with DAAs; (2) HCV reinfections reported in high-risk groups such as HIV-coinfected individuals, those with mental illness, and drug users; in Europe, for example, more than 50% of new HCV infections occur among drug users [18,19,20,21]; and (3) the onset of HCC despite achieving SVR after DAA treatment [22,23], keeping the debate on the effectiveness of DAA treatment in the prevention of hepatocarcinoma current.

In this review, after providing an overview of the characteristics of HCV and the defense mechanisms induced in the host, we will review the progress made and obstacles encountered in the development of anti-HCV vaccines.

## 2. Hepatitis C General Aspects

**Figure 1:**
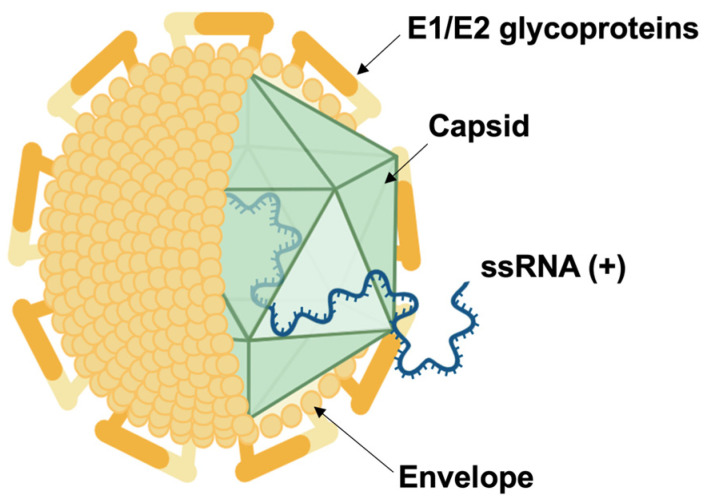
Hepatitis C virus (HCV) structure. Enveloped HCV virion comprises E1/E2 dimers. The viral genome consists of a single positive-sense RNA molecule enclosed in an icosahedral capsid composed of core proteins. Created with BioRender (https://www.biorender.com/, accessed on 19 July 2024).

HCV is a positive-strand enveloped RNA virus belonging to the genus *hepacivirus* of the *Flaviviridae* family. Its genome is approximately 9600 nucleotides in length and is translated into structural and non-structural proteins in the host cell [24]. The three structural proteins are core, Envelope 1 (Env1), and Envelope2 (Env2), while the non-structural proteins are p7, NS2, NS3, NS4A/B, NS5A, and NS5B (Figure 1) [25].

Some of these proteins are involved in different viral replication phases and play a crucial role in B- and T-cell immune responses (see below). Conversely, the 5′ and 3′ untranslated regions (5′-3′ UTR) influence viral replication but seem not to impact the immune response. The RNA-dependent RNA polymerase, NS5B, lacks proofreading activity and introduces many mutations during the viral replication cycle [26]. A study demonstrated that the *in vivo* mutation rate of HCV is 1.15 ± 0.29 × 10^−4^ [27], which is similar to the mutation rate observed in other RNA viruses [28]. This high error rate leads to fast evolution and genetic heterogeneity and generates a “quasispecies” population whose genetic variability can be appreciated by Next Generation Sequencing (NGS). Longitudinal studies carried out by NGS revealed that the mutation rate differs according to the region considered. For example, the UTR remains almost conserved, while Hypervariable Region 1 (HVR1) shows multiple variants, diversifying overtime to escape the host immune response [29] and to establish a chronic infection.

The high genetic variability has led to the phylogenetical classification of HCV into eight distinct genotypes (GTs). The core-E1 region, or NS5B region, is commonly used to determine HCV genotypes [30]. A difference of 30% or more at the nucleotide level distinguishes the genotypes [31]. Subtypes of genotypes have a sequence diversity of less than 15% [31]. All genotypes are indicated by Arabic numbers. The first isolated clone by Choo et al. was indicated as subtype 1a [32,33]. GT4 and GT6 are the most variable, with 18 and 29 genotypes, respectively [34,35]. The subtypes, which are indicated by a letter after the Arabic number, are classified by sequencing the core-E1 region from nt position 869 to 1292 of the H77 reference sequence [accession number AF09606] [36].

GT1 is diffused worldwide, representing 46% of all HCV infections [35]. It accounts for 75% of HCV infections in North America [37]. GT3 is the second most prevalent genotype (30% of HCV infections), mainly found in South Asian countries such as India, Bangladesh, Pakistan, Myanmar, Nepal, and Thailand, and among people who inject drugs (PWID) in European countries [38]. GT4 is mainly observed in Africa but has spread to European countries among PWID. GT5 is confined to Southern Africa, and GT6 is prevalent in Southeast Asia, particularly in Laos and Vietnam [39]. GT7 is rarely observed in Central Africa [40]. Recently, a new HCV genotype, GT8, has been identified in Punjab, India [41]. Moreover, full genome analysis has evidenced the presence of recombinant forms such as 1b/2k observed among IV drug users in St. Petersburg, Russia [42,43] or the intra-genotype 1a/1b recombinant form described in Peru [44]. Further information can be found on the Hepatitis Virus Database (http://hcv.lanl.gov/content/index, accessed on 19 July 2024) [34].

GT2 is another significant genotype of HCV. It is less prevalent globally but has distinct epidemiological characteristics. GT2 is often found in West Africa, southern Italy, and Japan [33]. It is associated with higher rates of SVR to antiviral therapy compared to other genotypes, particularly when treated with DAAs [45]. The relatively stable genetic structure of GT2, along with its responsiveness to treatment, makes it a key focus for epidemiological and clinical studies. GT2 infections are generally considered to have a better prognosis and treatment outcome compared to other genotypes, contributing to a more favorable public health perspective in regions where this genotype is predominant.

## 3. B-Cell Response

**Figure 2:**
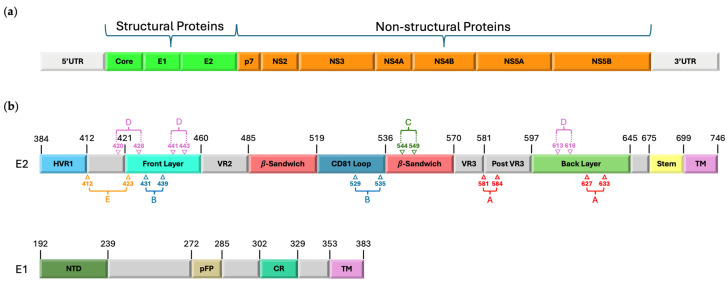
Schematic representation of the Hepatitis C virus (HCV) genome. (**a**) The figure shows the organization of genes encoding the structural (Core, E1, and E2) and non-structural (p7, NS2, NS3, NS4A, NS4B, NS5A, and NS5B) proteins of the virus. Structural proteins are indicated in green, and non-structural proteins are indicated in orange. (**b**) E1 and E2 protein segments are shown in detail, with boxes representing the different functional regions and their respective amino acid residue numbers in black. Additionally, the antigenic domains are mapped on the E2 protein as follows: A (red), B (blue), C (green), D (purple), and E (orange). HVR1, Hypervariable Region 1; VR2, Variable Region 2; VR3, Variable Region 3; Post VR3, Post Variable Region 3; TM, Transmembrane Domain; NTD, N-terminal Domain; pFP, putative Fusion Peptide; CR, Conserved Region.

The humoral response by B cells appears shortly after HCV infection but is inadequate to fully control the infection. The neutralizing antibody (nAb) response targets the envelope glycoproteins (GPs) E1 and E2, although E1 is less immunogenic compared to E2 [46]. Most nAbs target E2 epitopes, followed by E1–E2 heterodimers and E1 glycoproteins. The GPs E1 (residues 192–383, shown in Figure 2) and E2 (residues 384–746, shown in Figure 2) are type I transmembrane proteins with poorly understood roles, but E1 harbors a putative fusion loop. The E1 and E2 proteins’ ectodomains each have five and eleven glycosylation sites, respectively [47]. The glycans are crucial for E1–2 stability and antigenicity.

E1 and E2 form a heterodimeric complex (E1–E2 complex) on viral surfaces, mediating receptor binding and viral entry into host cells via interactions with CD81, scavenger receptor class B type 1 (SR-B1), and other molecules. After this interaction, claudin-1 (CLDN1), along with other claudin molecules, the tight junction (TJ) protein of hepatocytes, and Occludin, facilitate the activation of the entry pathway. In this manner, the envelope glycoproteins E1 and E2 are responsible for virus attachment and receptor-mediated endocytosis [48,49]. Antibodies against E1 and E2 are produced in the early phase of viral infection, but their levels remain low during chronic infection [50,51]. Vaccination studies in chimpanzees with E1–E2 proteins suggest their potential protective value, although antibody levels decline rapidly in HCV patients [52].

HCV infection can directly stimulate B cells via E2 binding to cell surfaces, leading to dysregulated cell proliferation independent of B-cell receptor (BCR) engagement [53]. As reported by a recent clinical trial of an HCV vaccine candidate, generating a T-cell response alone is insufficient for providing vaccine protection to high-risk individuals (NCT01436357). Therefore, stimulating a neutralizing antibody (nAb) response through vaccination is a priority in HCV vaccine research. There are five antigenic domains (A–E) identified by antibody and epitope mapping methods within aa residues 412–633 of the E2 protein.

Using the phage-display method, five discontinuous epitopes called Antigenic Regions (ARs) are recognized in the E2 or E1–E2 complex. The E2 neutralizing face (E2 NF) is found to be accessible on the viral surface and recognized by many cross-nAbs [54]. NF, overlapping with the CD81 binding site, is highly conserved and immunogenic, crucial for broad neutralizing antibodies bnAb like A3C [55]. However, bnAbs targeting E2 NF are often insufficient in quantity for protective levels due to molecular features in HCV env, including immunodominant regions like HVR1 and others, potentially competing with E2 NF in boosting B-cell responses [46,56].

The antibody response against HCV can be classified into three categories: broadly neutralizing, strain-specific, and non-neutralizing antibodies with distinct epitopes. Glycosylation sites on E2 regulate epitope accessibility, with conserved core regions contrasting with surface-exposed loops and HVR regions. The immune response in acute and chronic infections primarily targets the immunologically pressured HVR region of E2. The antigenic domain A (and AR1) primarily induces non-neutralizing antibody response [57]. Several neutralizing Abs against the domain B-E in the E2 core structure (domains B-E) mostly target the highly conserved flexible CD81 binding site, the front layer, and the CD81 binding loop (residues 519–535), inhibiting cell entry [54].

Domain E, corresponding to the segment adjacent to HVR1 (aa 412–423), is also designated epitope I or AS412 and contains highly conserved neutralizing epitopes. A mouse monoclonal antibody (AP33) specific for the residues 412–423 shows broad neutralization against HCV virions bearing E1–E2 from major HCV genotypes 1–6 [58]. Understanding antibody–epitope interactions is crucial for designing HCV vaccines.

Domain D corresponds to the region encompassing residues 420–428 and 441–443, with contributions from residues 613–616; it is also designated AS432. Neutralizing antibodies against domain D inhibit E2 binding to CD81 interaction and block virus entry [59].

Domain B of HCV E2 is highly immunogenic, containing overlapping epitopes inducing potent neutralizing antibodies, with escape mutants observed (e.g., D431G, A439E) [60].

Domain C, which mediates broad neutralization, is located in part at residues 544–549 in the central beta sandwich of the E2 [46].

Broad neutralization is also mediated by antigenic regions 4 and 5, formed at the interface of E1–E2 residues. Specific sites AS108, AS112, and AS146 have been identified within AR5 [61].

Antibodies targeting HVR1, encompassing the initial 27 amino acids (384–410) at the N terminus of the E2 region and forming part of the AR3 epitope, are found in the majority of HCV-infected individuals. In animal models, these antibodies may drive the replication of viral variants that evade recognition by pre-existing antibodies [62]. In animal models, antibodies against HVR1 appear to elicit a stronger response compared to those against recombinant HCV E1E2 proteins. However, over time, these HVR1 antibodies drive the replication of viral variants that the existing antibody response fails to recognize [63,64]. Another study demonstrated that neutralizing antibodies during chronic infection may not neutralize new HVR1 variants [65]. HVR1 deletion significantly enhanced HCV neutralization sensitivity, increasing it by three orders of magnitude and indicating that eliminating HVR1 maximizes neutralization sensitivity [66,67]. Studies have shown that high-titer antibodies can effectively suppress viral replication, preventing the emergence of escape mutants, likely due to the low fitness of these mutants [68]. This finding underscores the importance of ensuring the efficacy of an HCV vaccine. It suggests that an effective vaccine should induce antibodies with a sustained titer and incorporate conserved epitopes to minimize the viral escape phenomenon.

Several factors can inhibit the nAbs activity, in addition to a genetic mutation that allows viral escape. There are various evasion strategies that negatively affect the neutralizing antibody response. These include glycosylation of the virus envelope, non-neutralizing antibodies, or virus-associated proteins, which can interfere with antibody-mediated neutralization by either masking neutralization epitopes or limiting access of neutralizing antibodies to their target epitopes [69,70,71]. It has been proposed that a segment of the E2 protein spanning amino acids 434–446 (epitopes II) contains epitopes associated with non-neutralizing antibodies. These antibodies can interfere with the neutralizing activities of antibodies directed at an adjacent E2 segment spanning amino acids 412–426 (epitopes I) [72,73].

This suggests that a possible vaccine must contain epitopes that stimulate the production of only some neutralizing antibodies in order to avoid negative interference.

Another limitation of the B cell response is its weak antibody protection against different HCV genotypes. Cross-reactivity among different genotypes is minimal, estimated at less than 10% [74,75]. Generally, the level of nAb in convalescent HCV patients is very low, which likely explains why nAbs from previous infections cannot protect against reinfection by different strains or genotypes [19,76]. Additionally, immunodominance can be influenced by genotype [77]. There are no specific epitopes associated with viral clearance; rather, viral clearance is linked to the presence of multiple nAbs targeting unique neutralizing domains [77,78].

## 4. Adaptive Immune Response T Cells

**Figure 3:**
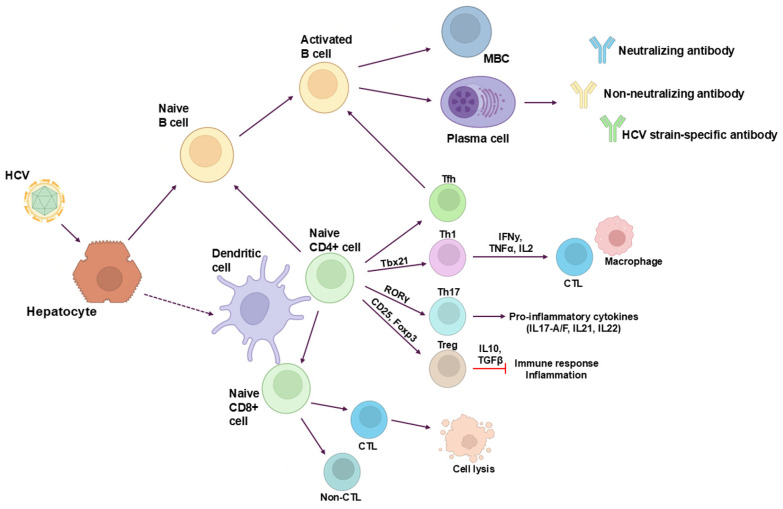
Adaptive immunity during HCV infection. Immune response involves activation of dendritic cells and other components of innate immunity, whereas B lymphocytes, CD4+ T cells, and CD8+ T cells are effectors of adaptive immunity. Abbreviations: Th1, T helper 1 cell; Th17, T helper 17 cell; Treg, T regulatory cell; Tfh, T follicular helper cell; MBC, memory B cell; CTL, cytotoxic lymphocyte. Created with BioRender.

T cells, both CD4+ and CD8+, play an important role in defending against pathogens by producing cytokines that can inhibit pathogen replication and by lysing infected cells (Figure 3). Rapid control of HCV infection is marked by the expansion of both CD4+ and CD8+ T cells in peripheral blood [79,80]. A weak T cell response, particularly CD4+ T cells, during the first six months is a strong predictor of persistent infection [81]. During the acute phase, the CD4+ T cell response is very broad, targeting mainly 10 classes of MHC class II-restricted epitopes primarily located in the non-structural regions NS3 and NS4 [82].

In the case of persistent HCV infection, the repertoire of CD4+ T cells decreases until it becomes undetectable [82,83]. Furthermore, CD4+ T cells express several inhibitory receptors, including programmed death-1 (PD-1), cytotoxic T-lymphocyte-associated protein 4 (CTLA-4), CD305, and CD200r [84]. The signaling from these inhibitory receptors influences the adaptive immune response, regulating the cytotoxic T lymphocyte (CTL) response and antigen-specific B lymphocytes. CD4+ T cells can differentiate into Th1, Th17, or Treg cells, each with distinct functions/activities influenced by stimuli received through their TCR and cytokine milieu produced during acute HCV infection [85]. Th1 cells, the main pro-inflammatory subset, express the transcription factor *T-bet* (Tbx21) and participate in cell-mediated immune responses by secreting IFN-γ, TNF-α, and IL-2, which stimulate macrophages and CTL functions [85]. Treg lymphocytes, expressing CD25 and Foxp3, suppress immune responses and inflammation to maintain self-tolerance, acting through direct cell–cell contact and the secretion of anti-inflammatory cytokines such as IL-10 and TGF-β [86]. Moreover, Treg lymphocytes are increased in individuals with HCV infection [87]. Th17 cells, characterized by ROR-γ expression and the secretion of IL17-A/F, IL21, and IL22 [85], are considered pro-inflammatory since they recruit other inflammatory immune cells. Th17 lymphocytes are implicated in the pathogenesis of other liver diseases of different etiologies, such as alcoholic hepatitis, primary biliary cirrhosis, and chronic hepatitis B [88]. The role of Treg in liver damage remains debatable, but their presence in chronic hepatitis C (CHC) liver suggests a potential role in immune response modulation. The correlation between Treg and both CTL and Th1 cells indicates that the inflammatory state in the liver may promote recruitment or differentiation towards a Treg profile. Tregs may help control effector lymphocytes (CTL presence and Th1); thus, the lack of association between CTLs and Th1 cells with the severity of hepatitis or fibrosis could be due to Treg-mediated inhibition of Th1 and CTL actions, thereby preventing liver damage [89].

Finally, the T follicular helper (Tfh) cells, a specialized subset of CD4+ T cells, play a pivotal role in the germinal center (GC) response, providing essential help to B cells for the production of effective antibody-mediated immune responses. Virus-specific Tfh cells have been detected in the blood of patients acutely infected by HCV [90]. In these patients, a correlation between the frequency of HCV-specific activated circulating Tfh (cTfh) cells and the production of plasma HCV NS4-specific antibodies has been reported, while in chronically infected HCV patients, cTfh cells seem to disappear [90].

In addition, E2-specific memory B cells (MBCs) from individuals who spontaneously resolved HCV infection were observed to peak early after infection (4–6 months), correlating with the expansion of activated cTfh cells expressing interleukin 21 (IL-21). This early activation of cTfh cells was associated with the production of monoclonal antibodies with fewer somatic hypermutations but effective neutralizing capacity, contributing to the spontaneous clearance of the virus. In contrast, chronically infected subjects exhibited a delayed expansion of E2-specific MBCs, which occurred in the absence of significant cTfh cell activation [91].

CD8+ T cells specific to HCV virus response are present at higher levels than CD4+ T helper cells in chronic HCV infection. They persist for decades and target multiple Class I epitopes.

CD8+ T cells are crucial for controlling HCV infection since they eliminate infected cells by releasing cytotoxic granules and expressing cell-death-inducing receptor ligands. They also inhibit viral replication through non-cytolytic mechanisms, such as IFN-γ and TNF-α secretion. However, in HCV chronic infection, CD8+ T cells may be functionally impaired regarding IFN-γ production, proliferation, cytotoxicity, and potential degranulation [92]. Furthermore, while the presence of CTLs in the liver helps inhibit viral replication, it can also lead to liver damage due to inflammation and increased cytokine production at the site of infection. The presence of intrahepatic Treg may suggest their regulatory function in mitigating liver damage by regulating the immune response [89].

The antiviral activity of CD8+ T cells can be blocked in several ways, including interference with antigen processing or the expression of Class I MHC, as demonstrated in hepatocytes *in vitro* [93], and the production of inhibitory cytokines by regulatory T cells [94]. Two main mechanisms can impair CD8+ T cell activity: (1) Mutational escape of Class I epitopes of HCV and (2) physiological exhaustion, which leads to the loss of their effector functions [95].

Viral escape is the primary mechanism used by HCV to evade CD8+ T cell antiviral activity. In chronically infected HCV patients, the viral genome exhibits nonsynonymous mutations that enable viral escape from CD8+ T cell recognition. This phenomenon is more frequently observed in class I restriction epitopes than in flanking sequences [96]. The nonsynonymous mutation rate is particularly pronounced during the first 12 years of HCV infection and then declines as the infection persists [97]. These selected nonsynonymous mutations associated with viral escape become fixed during chronic infection [98]. The decrease in CD4+ T helper cells and the limited antigen receptor repertoire in CD8+ T cells hinder the generation of new CD8+ T cells capable of counteracting the new mutant epitopes [99]. Additionally, the persistence of HCV is not necessarily dependent on the mutational escape of Class I epitopes. The rate at which Class I epitopes develop mutations varies significantly across patients, with estimates ranging from 25% to 50% [100]. CD8+ T cells continue to recognize intact epitopes but tend to become exhausted and terminally differentiated over time [101]. These virus-specific CD8+ T cells exhibit prolonged expression of several inhibitory receptors, such as PD-1, CTLA-4, T cell immunoglobulin, and nucleic domain-containing molecules 3 (TIM-3) [102]. This exhausted state is marked by increased inhibitory receptor expression, disrupted metabolic processes, reduced survival capacity, and distinct transcriptional and epigenetic alterations. T cells are pivotal in determining whether an acute HCV infection resolves spontaneously or progresses to chronicity.

Finally, there are unconventional T cells (UTCs), a heterogeneous group of T cells known for their rapid response to specific pathogen antigens. Chronic HCV infection causes dysfunction in several subsets of UTCs. During HCV infection, the UTC phenotype is altered, and this alteration persists even after the clearance of chronic HCV by direct-acting antivirals (DAAs). However, the response of UTCs to acute HCV infection is not fully understood. Notably, the frequency of mucosal-associated invariant T (MAIT) cells decreases during HCV infection. Key subsets of UTCs include MAIT cells, γδ T cells, double-negative T cells (DNT cells), and CD1d-restricted NKT cells [103]. MAIT cells are found in mucosal tissues, the liver, and peripheral blood [104,105].

A reduction in MAIT cells has been observed during HCV infection, with circulating MAIT cells exhibiting an exhausted phenotype and an altered response to bacterial challenge [106,107]. An altered phenotype is also observed in γ/δ T cells, which become less efficient in cytokine production [108,109]. DNTs respond to HCV infection by producing more than 30 inflammatory mediators. The UTC activation may be driven by inflammatory cytokines, though it remains to be determined whether they play a role in controlling liver damage.

The adaptive immune response during HCV infection is illustrated in Figure 3.

The vast genetic diversity and the propensity to evade the host immune responses represent the main obstacles in the development of an HCV vaccine. However, the evidence that approximately 25% of HCV acutely infected individuals spontaneously resolve the infection and that, if reinfected, 80% of these individuals clear viremia indicates that adaptive immunity against HCV can be induced, and although it is not sterilizing, it can protect against chronic infection [110].

These data have stimulated the study of the protective immune responses that are able to control HCV during infection. Early studies on protective immunity pointed to the important role of cellular as well as humoral immune responses in the control of HCV infection.

Strong, broad, specific, vigorous, and multispecific CD4+ T helper cells and CD8+ cytotoxic T cell responses targeting the non-structural protein of HCV have been detected during the early stage of the infection in people who spontaneously resolve infection [111,112]. Similar results were obtained in chimpanzees, and in this model, depletion of CD4+ or CD8+ T cells in the reinfected animals resulted in the persistence of viremia [113,114].

Concerning the humoral immune response, early studies showed that *in vitro* neutralization of HCV with a rabbit hyperimmune serum raised against HVR1 induced protection against the homologous HCV infection in chimpanzees but not against the emergence of neutralization escape mutants [115]. In addition, vaccination with E1 and E2 glycoproteins could protect chimpanzees from intravenous infection with the autologous virus [116].

The definitive evidence that antibodies could protect from HCV persistent infection in humans was obtained in studies in which, using autologous HCV pseudoparticles (HCVpp), a correlation was demonstrated between the presence of neutralizing Abs in the acute phase of the infection and both control of viremia and spontaneous resolution [117,118]. In addition, using a GT1 HCV pseudoparticle library, it was reported that control of HCV infection is associated with more rapid development of a broad nAb response, independent of the infection viral genotype [119].

The development of prophylactic HCV vaccines that have been evaluated in clinical studies followed two main approaches that were distinguished for their ability to preferentially induce T cell-mediated or humoral immune responses. Induction of T cell responses by the vaccine was achieved through the delivery of non-structural HCV antigens by viral vectors. Induction of HCV-specific antibodies by immunization with recombinant envelope proteins.

## 5. B Cell Vaccine

One of the first vaccine candidates against HCV consisted of the GT1a E1 and E2 glycoproteins purified from mammalian Chinese hamster ovary cells. Vaccination of seven chimpanzees with E1E2 followed by intravenous HCV infection showed that the five vaccinees that developed high antibody titers were sterilized [116] against homologous virus infection, whereas the two chimpanzees with low anti-E1E2 antibodies developed a milder disease.

Reexamination of the sera in the protected chimpanzees revealed that four of the five protected chimpanzees had significant titers of cross-neutralizing antibodies reacting with HCVpp, representing genotypes 1a, 4a, 5a, and 6a [120]. However, when nine chimpanzee vaccinees were challenged with the GT1a HCV-H strain, although none of the vaccinated animals was protected against the acute infection, all but one resolved the infection, demonstrating that the vaccine was able to protect against chronic infection with either homologous or heterologous HCV GT1a [121].

Clinical testing of the E1/E2 glycoproteins adjuvanted with MF59C.1 (a squalene oil/water (o/w) emulsion without the presence of additional immunostimulators [122]) in a phase I randomized, double-blind, placebo-controlled dose-escalation study demonstrated that the vaccine was well tolerated and that all immunized volunteers developed antibodies against the glycoproteins gpE1/gpE2 and strong T-helper responses to the vaccine [123].

Investigation of the breath of the neutralization response elicited by vaccination in eight healthy immunized volunteers revealed that all immune serum samples neutralized HCVpp expressing the closely related GT1a, the heterologous GT1b, and the more distantly related GT2a strain, albeit less efficiently [124].

Testing of cross-neutralizing activity against representatives of all major genotypes of HCV showed that at least 1 of the 16 vaccinees tested developed broad cross-neutralizing antibodies against all known major genotypes of HCV, although with different efficiencies. Although observed in a minority of vaccinees, these results proved the key concept that a vaccine derived from a single strain of HCV can elicit broad cross-neutralizing antibodies [125].

Important insights into the characteristics of antibody responses elicited by the recombinant E1E2 glycoproteins utilized in the clinical trial were obtained by the analysis of vaccinated volunteers and after vaccination of non-human primates and mice. In humans and in the two animal models, the dominant antibody responses elicited by vaccination were directed against the non-neutralizing epitopes E1 N-terminus and E2 HVR1. In contrast, neutralizing antibody responses to the known antigenic sites AS412, AS434, and AR3 were relatively rare. In humans and in NHPs, the nAb antibodies elicited by the vaccine were mainly directed against AR3, but their level in the blood was insufficient for broad neutralization [126].

A comparison of the immunoreactivity and antigenicity between the recombinant HCV GT1a strain H77C envelope glycoprotein heterodimer gpE1/gpE2 and the recombinant gpE2 alone, derived from an infectious molecular clone (H77C), revealed that gpE1/gpE2 has a stronger binding affinity for a wider range of bnAbs than gpE2 alone. Moreover, gpE1/gpE2 induced a broader cross-neutralizing antibody response in animal models, particularly against more heterologous, non-1a genotypes. These findings support the inclusion of gpE1/gpE2 in the development of an HCV vaccine [127].

## 6. B Cell Vaccine Optimization by Antigen Modification, Adjuvants and Formulation

The analysis of the characteristics of the antibody response elicited by the E1/E2 vaccine underlined the need to enhance the immunogenicity of the vaccine, possibly through the modification of the antigens, adjuvants, and formulation.

Based on the evidence that HVR1 is immunodominant and can restrict access to many neutralizing antibodies, it has been proposed that gpE1/gpE2 lacking this domain could be a better vaccine antigen to induce broadly neutralizing antibodies [128]. A comparison of the immunogenicities of WT and ΔHVR1 gpE1/gpE2 in mice showed that the removal of HVR1 adversely affected the immunogenicity of the glycoprotein. Although ΔHVR1 gpE1/gpE2 induced similar titers of antibodies, ∆HVR1 gpE1/gpE2 immune serum displayed lower neutralization activity towards the homologous virus. However, cross-neutralization of G3a, G4a, and G5a viruses was similar for immune sera from both groups, suggesting that the removal of HVR1 does not affect the generation of cross-Nabs [128].

Another approach to focusing the immune response on highly conserved nAb epitopes consisted of the deletion of the three variable regions (HVR1, HVR2, and VR3) from a recombinant subdomain of E2, resulting in the generation of a soluble E2 core domain (residues 384–661) that maintained CD81 binding and recognition by human monoclonal antibodies [129]. Immunization of guinea pigs with the high molecular form of the ∆123 antigen in the ISCOMATRIX adjuvant elicited distinct antibody specificities with potent and broad neutralizing activity against all seven HCV genotypes [130].

Optimization of the E2 core protein in terms of antigenicity and stability was obtained by mutation of seven cysteine residues in the ∆123 [131].

Since glycosylated asparagine (Asn) residues in E2 play an important role in the masking of epitopes recognized by broadly neutralizing antibodies, another approach to enhance envelope glycoprotein immunogenicity consisted of the modification of glycosylated sites of the proteins [69,132].

Deletion of N-glycans through site-directed mutagenesis and the addition of immune activator CpG during DNA vaccination induced the highest cellular immune response compared to the WT E1E2. The anti-serum induced by mouse immunization with the mutated E1/E2 proteins effectively neutralized the infection of cell-cultured HCV and HCV pseudoparticles (HCVpp, genotypes 1 to 7) in Huh-7.5.1 hepatocytes [133].

In addition, the soluble E2 produced in insect cells, which impart mainly paucimannose-type glycans, was more immunogenic compared to the soluble E2 produced in mammalian cells, which confer mostly complex- and high-mannose-type glycans [134]. An increase in the immunostimulatory capacity of a sE2 variant showing the addition of a potential N-linked glycosylation site and reduced CD81 binding activity (sE2F442NYT) was observed in mice immunized with an E1/sE2F442NY-mRNA lipid nanoparticle preparation [135].

The selection of an appropriate adjuvant ideally capable of inducing robust, long-lasting, humoral, and cell-based immune responses is needed for the development of an efficacious HCV vaccine.

To further improve neutralizing antibody and T-cell responses, the immunogenicity of HCV rE1/E2 combined with different adjuvants was evaluated in mice. H77 rE1/E2 formulated with Alum/MPLA, MF59, c-di-AMP, or archaeosomes (liposomes based on archaeal lipids [136,137]) induced neutralizing antibodies able to prevent the entry of HCVpp expressing GT1a (H77) in an *in vitro* neutralization assay [138]. Administration of the antigen in combination with c-di-AMP or archaeosomes induced more robust T-cell responses compared to those elicited by the Ag with Alum/MPLA. rE1/E2 adjuvanted with MF59 did not induce T-cell responses. Further studies on different formulations of sulfated S-lactosylarchaeol (SLA) archaeosomes, whereby E1/E2 were encapsulated within or admixed with SLA archaeosomes, revealed that the simpler admixed archaeosome formulation SLA (Enc) generated strong levels of HCV neutralizing antibodies and polyfunctional antigen-specific CD4+ T cells producing multiple cytokines such as IFN-γ, TNF-α, and IL-2 [139].

Another approach to improving the immunogenicity of HCV candidate vaccines consisted of their presentation on nanoparticles. Self-assembled nanoparticles displaying sE2 on the surface were obtained after the fusion of sE2 with a ferritin unit. The sE2 moiety on the sE2-ferritin nanoparticle had nearly natural conformation and better affinities than the unfused sE2 to neutralize antibodies, receptors, and patient serum. sE2-ferritin was more potent than sE2 in inducing broadly neutralizing anti-HCV antibodies in immunized mice [140].

Multiple NP platforms were used to display an optimized version of the sE2 from HCV GT1a and GT6a, and their antigenicity and immunogenicity were tested. After redesigning variable region 2 in a truncated form (tVR2) on E2 cores derived from GT1a and GT6a, the E2 cores were displayed on ferritin (24-mer), E2p (60-mer), and I3-01 (60-mer) nanoparticle platforms. The E2 nanoparticles demonstrated high yield, high purity, and enhanced antigenicity with respect to individual E2 cores. In addition, nanoparticles induced more effective NAb responses than soluble E2 [141].

Recently, soluble E2E1 trimers were engineered for HCV vaccine development through the permutation of genes encoding the E1 and E2 subunits. The resulting E2E1 trimers were presented on two-component nanoparticles, a design that facilitated the creation of nanoparticle cocktails and mosaic nanoparticles displaying multiple E2E1 trimers simultaneously. These mosaic nanoparticles elicited a significant breadth of neutralizing antibodies after just two immunizations [142].

Virus-like particles (VLPs) constitute another approach to the development of HCV vaccines. Several HCV VLP-based vaccines have been shown to elicit cross-reactive nAb and T cell responses in different animal models, including mice and macaques, and to confer partial protection in chimpanzees [143,144].

More recently, a quadrivalent HCV VLP vaccine containing particles of genotypes 1a, 1b, 2a, and 3a has been developed, and it has been shown that it produced strong antibody and T cell responses in mice and in a pre-clinical large animal model. Indeed, intradermal vaccination of Landrace pigs with quadrivalent VLP generated durable HCV multi-genotypic neutralizing antibodies and CMI responses [145,146].

Finally, it has been reported that replication-deficient culture-generated HCV (HCVcc) more efficiently induced the production of anti-E1 and anti-E2 antibodies compared to the recombinant E2 glycoproteins in a rodent model [147]. In addition, immunization of non-human primates with a chimeric GT2a replication-deficient HCV, in combination with an adjuvant, induced cellular responses and antibodies against HCV structural proteins that cross-neutralized HCVpp (genotypes 1a, 1b, and 2a) and HCVcc (genotypes 1a, 1b, 2a, and 3a) [148].

## 7. T Cell Vaccine

The concept of a prophylactic T-cell vaccine was first demonstrated in a chimpanzee challenge model [149].

Vaccination with adenoviral vectors and plasmid DNA coding for the non-structural region (NS3–NS5) of the HCV GT1b protected chimpanzees from acute hepatitis induced by challenge with a heterologous GT1a virus differing from the vaccine sequence by more than 13% at the amino acid level. In chimpanzees successfully vaccinated, HCV-specific T cells emerged earlier, retained greater functionality, and persisted at higher frequencies for longer periods following the HCV challenge compared to those in mock-vaccinated chimpanzees. The vaccine-induced T cells exhibited higher expression of CD127, lower levels of PD-1, and demonstrated multifunctionality compared to infection-induced T cells [150].

In humans, two T-cell prophylactic vaccines have been tested for immunogenicity. The first one consisted of the HCV core protein and ISCOMATRIX adjuvant (HCV Core ISCOMATRIX TM vaccine), while the second vaccine delivered genes encoding NS3, NS4, NS5A, and NS5B within a replicative defective simian adenoviral vector (CHAd3) and a modified vaccinia Ankara (MVA) vector.

The safety, tolerability, and immunogenicity of the HCV Core ISCOMATRIX TM vaccine in healthy individuals were evaluated in a Phase I placebo-controlled dose escalation clinical study. The vaccine was immunogenic, safe, and well-tolerated. Humoral immune responses occurred in all subjects, and T cell cytokines were detected in 7 of the 8 participants, while CD8+ T cell responses were only detected in two of the eight participants receiving the highest dose of HCV core protein, suggesting that the limited CD8+ T cell immunogenicity of the vaccine could be improved by the use of larger HCV antigens [151].

The development of a viral vector HCV vaccine for humans required preliminary studies to identify suitable vaccine carriers. Thus, a large collection of replication-defective adenovirus vectors was generated and screened for several characteristics, including neutralization by human sera, the ability to grow in human cell lines already approved for clinical studies, and immunological potency. This led to the selection of two of the most potent chimpanzee adenovirus vectors (ChAd), ChAd3 and ChAd 63, allowing the design of a vaccination strategy based on heterologous prime-boost modality with serologically distinct ChAd to maximize immunization strength [152]. The first highly immunogenic HCV T cell vaccine used a heterologous prime/boost vaccination regimen based on priming with ChAd3 and boosting with MVA encoding the NS3, NS4, NS5A, and NS5B proteins of HCV GT1b with an inactivating mutation in the HCV polymerase (ChAd3-NSmut/MVA-NSmut) [153].

Assessment of the safety and immunogenicity of the vaccine in a phase I trial demonstrated that ChAd3-NSmut priming followed by MVA-NSmut boosting induced very high levels of both CD8+ and CD4+ HCV-specific T cells targeting multiple HCV antigens in healthy volunteers [154].

Sustained expansion of HCV-specific effector memory T cells (Tem) was detected, and memory T cells showed an improvement in proliferation and polyfunctionality over time. The T cell responses induced by the vaccine 1b genotype cross-reacted with antigens from heterologous HCV genotypes, although the frequencies of IFN-γ SFCs specific for GT1a were reduced by about 30% and those specific for GT3a and GT4a by about 70%.

The safety and efficacy of the ChAd3-NSmut/MVA-NSmut vaccine were assessed in a phase 1–2 randomized, double-blind, placebo-controlled trial conducted between 2012 and 2018. The trial included HCV-uninfected individuals at high risk for HCV infection, who were randomly assigned to receive an intramuscular injection of either the ChAd3-NSmut vaccine (2.5 × 10^10^ viral particles) on day 0 and the MVA-NSmut vaccine (1.8 × 10^8^ plaque-forming units) on day 56 (vaccine group) or a saline placebo on the same days (placebo group). A total of 548 volunteers were monitored for 20 months post-enrollment and for 9 months post-infection. Of these, 202 participants in the vaccine group and 199 in the placebo group were followed until either chronic infection or trial completion. A total of 14 participants in each group developed chronic HCV infection, indicating that the vaccine regimen did not prevent chronic infection. However, the vaccine-induced HCV-specific T-cell responses reduced peak HCV RNA levels and did not result in serious adverse events [155].

Finally, targeting Tfh cells represents a novel strategy in the development of HCV vaccines aimed at enhancing the quality of the humoral immune response. Recent vaccine designs incorporate antigens that specifically stimulate Tfh responses. For instance, viral vector-based vaccines have shown the potential to induce robust Tfh responses, leading to the generation of potent bnAbs against HCV [156]. Additionally, studies have demonstrated that virus-specific CD4+ T cells in both acute and chronic HCV infections exhibit functional and phenotypic characteristics of Tfh cells, suggesting that targeting these cells could be a promising approach for vaccine development [90]. This approach addresses the challenge posed by HCV’s high mutation rate, aiming to provide long-lasting immunity and protection across diverse viral strains.

## 8. Conclusions

HCV infection represents a relevant public health problem, causing chronic infection in 75% of cases. Therapy with pangenotypic DAA leads to the eradication of the virus in >90% of cases; however, it does not eliminate the possibility of developing hepatocellular carcinoma (HCC). Furthermore, reinfections cannot be avoided, and this is a significant problem, especially in some categories, such as drug users. Therefore, the development of a vaccine is still a main subject in HCV infection management. However, this is a great challenge due to the high genetic variability and mutational escape of the virus. At present, the development of prophylactic HCV vaccines that have been evaluated in clinical studies followed two main approaches that were distinguished for their ability to preferentially induce T cell-mediated or humoral immune responses. The most advanced B cell-targeting vaccine consisted of the E1/E2 heterodimer. The vaccine induced neutralizing antibodies, but their level in the blood was insufficient for broad neutralization.

Reverse Vaccinology and Structure-Based Vaccine Design have been used to optimize the response to the vaccine, but the results are not yet definitive [157,158,159].

Even vaccines capable of stimulating the T response with broader efficacy against different HCV genotypes have not given satisfactory results. In humans, two T-cell prophylactic vaccines have been tested. The first one consisted of the HCV core protein and ISCOMATRIX adjuvant, while the second vaccine delivered genes encoding NS3, NS4, NS5A, and NS5B within a replicative defective simian adenoviral vector (CHAd3) and MVA vector. In the first case, the CD8+ T cell response was modest, so much so that in one patient, no appreciable response was observed, while in the second case, the CD8+ T cell response was more sustained; however, a non-negligible part of the subjects to whom the vaccine was administered (14/202) developed a chronic HCV infection, suggesting that the success of a future protective vaccine may rely on its ability to engage both humoral and cellular immune responses.
